# A key to species of the genus *Gastroserica* Brenske of the China (Coleoptera, Scarabaeidae, Sericini), with the description of two new species and two new records for China

**DOI:** 10.3897/zookeys.139.1702

**Published:** 2011-10-25

**Authors:** Wan-Gang Liu, Dirk Ahrens, Ming Bai, Xing-Ke Yang

**Affiliations:** 1 Key Laboratory of Zoological Systematics and Evolution, Institute of Zoology, Chinese Academy of Sciences, Box 92, No. 1, Beichen West Road, Chaoyang District, Beijing, 100101, P.R. China; 2 Graduate School, Chinese Academy of Sciences, Yuquan Road, Shijingshan, Beijing, 100039, China; 3 Zoologisches Forschungsmuseum A. Koenig, Adenauerallee 160, 53113 Bonn, Germany

**Keywords:** Coleoptera, Scarabaeidae, Sericini, *Gastroserica*, China, new species, key

## Abstract

Based on a revision of the material housed in Chinese collections and a key to species of *Gastroserica* of China is provided. Two new species are described, habitus photographs, and illustrations of the genitalia are given: *Gastroserica nigrofasciata* **sp. n.** (from China: Guangxi and Guizhou Prov.), *Gastroserica yunnanensis* **sp. n.** (from China: Yunnan Prov.). Besides, illustrations of the genitalia of species mentioned in the key are provided. Additional distribution records of the *Gastroserica* species including an updated distribution map are given.

## Introduction

The genus *Gastroserica* was established by Brenske (1897) based on the character of antenna (club with four antennomeres in males, but with three or four in females) and the obsolete anterior angles of pronotum. Currently, the genus is defined by the club with four antennomeres in males, but with three or four in females; mentum oblate; anterior angles of pronotum not produced; pygidium long, apically produced, not completely covered by elytra.

The species of the genus from Asiatic mainland were revised by Ahrens (2000). Since that contribution, eight additional species were described (Ahrens 2002, 2007, Ahrens and Pacholátko 2003). So far, there are 35 species described, all occurring in East and Southeast Asia. From China, so far 20 species are recorded. In this paper, we survey the material hold in Chinese institutional collections. This rich material contained two new taxa that are described herein. In addition to published data, new distribution data including two new species records from China are given, along with an updated distribution map and a key to the species of *Gastroserica* occurring in China.

## Material and methods

All descriptions and measurements were made under an Olympus SZ 61 microscope, and all figures were made with a digital camera (Nikon D300S) attached to a stereomicroscope (Zeiss Discovery V12) and Helicon Focus 5.1 software. The distribution map was made with ArcGIS9.2 and Adobe Photoshop CS3. Type specimens and other material are deposited in the Institute of Zoology, Chinese Academy of Sciences, Beijing, China (IZAS), College of Life Sciences, Sun Yat-sen University, Guangzhou, China (LSSYU), National Museum Prague (Natural History), Czech Republic (NMPC), Zoologisches Forschungsmuseum A. Koenig Bonn, Germany (ZFMK).

## Taxonomy

### Key to the genus Gastroserica of China

**Table:** 

1	Labroclypeus widest at middle, lateral margins more or less distinctly narrowed towards base, or subparallel. Pronotum with or without longitudinal median impressions	2
–	Labroclypeus widest at base, lateral margins narrowed anteriorly. Pronotum without longitudinal median impression	18
2	Disc of pronotum with shallow longitudinal median impression or additionally with a transverse impression behind the middle	3
–	Disc of pronotum without a shallow longitudinal median impression	9
3	Disc of pronotum with a transverse impression behind the middle	4
–	Disc of pronotum only with a shallow longitudinal median impression	7
4	Eyes strongly protruding, labroclypeus very narrow (ratio maximal width of head including eyes/ width of labroclypeus: 1.8–1.9). Pygidium strongly conical. Pronotum with distinct median transversal impression	*Gastroserica impressicollis* (Fairmaire, 1891)
–	Eyes weakly protruding, labroclypeus relatively wide (ratio maximal width of head including eyes/ width of labroclypeus: 1.48). Pygidium moderately convex. Median transversal impression on pronotum weak	5
5	Pronotum with a distinct longitudinal median impression on the disc and a round median impression behind the disc	*Gastroserica hubeiana* Ahrens, 2000
–	Pronotum with only a longitudinal median impression on the disc	6
6	Frontoclypeal suture indistinctly impressed. Pronotum with several setae in the longitudinal median impression. Aedeagus with a long apophysis on the left side of the apical phallobase, and the dorsal lobe of left paramere split from basal lobe at base (Fig. 2 J–L)	*Gastroserica kucerai* Ahrens & Pacholátko, 2003
–	Frontoclypeal suture distinctly impressed. Pronotum with few setae in the longitudinal median impression. Aedeagus without a long apophysis on the left side of the apical phallobase, and the dorsal lobe of left paramere split from ‘basal’ lobe at middle of paramere’s length (Fig. 2 G–I)	*Gastroserica sichuanana* Ahrens, 2000
7	Punctures on pronotum fine, simple and scarcely scattered; median longitudinal impression fine and superficial	8
–	Pronotum strongly and densely punctate, between punctures additionally with microscopic fine punctures; median longitudinal impression robust	*Gastroserica sulcata* Brenske, 1897
8	Both parameres simple, the left one pointed externally (Fig. 4 P)	*Gastroserica bicolor* Nijima & Kinoshita, 1923
–	Both parameres bifurcate, the left one pointed medially (Fig. 3 A–C)	*Gastroserica herzi* (Heyden, 1887)
9	Aphophysis of phallobasis short and wide, apically lobiform and rounded (Fig. 3 J–L)	*Gastroserica haucki* Ahrens, 2000
–	Aphophysis of phallobasis long and sharply pointed	10
10	Lateral margins of labroclypeus strongly reflexed	*Gastroserica angustula* Brenske, 1897
–	Lateral margins of labroclypeus weakly reflexed	1
11	Disc of pronotum with punctures of equal size bearing minute setae. Apex of elytra sclerotized, without a rim of minute microtrichomes. Apical apophysis of phallobasis apically without a sharp hook	12
–	Disc of pronotum with large punctures bearing each a robust seta and being twice as large as smaller ones. Apex of elytra with a membraneous rim composed of minute microtrichomes. Apical apophysis of phallobasis apically with a sharp hook	17
12	Ocular canthus short, ratio length of ocular canthus/ ocular diameter: < 0.33	13
–	Ocular canthus long, ratio length of ocular canthus/ ocular diameter: > 0.42	14
13	Apophysis of phallobasis long, exceeding the parameres in length (Fig. 3 M–O)	*Gastroserica fanjingensis* Ahrens, 2000
–	Apophysis of phallobasis very short, distinctly shorter than the parameres (Fig. 4 D–F)	*Gastroserica nikodymi* Ahrens, 2000
14	Metatibia externally along the middle very densely and coarsely punctate. Metatasromere 1 slightly shorter than the two following tarsomeres combined. Colour of pronotum and elytra yellowish brown, sometimes with dark spots	*Gastroserica asulcata* Ahrens, 2000
–	Metatibia externally along the middle with moderately to feebly punctate. Metatasromere 1 distinctly longer than the two following tarsomeres combined. Colour of pronotum and elytra dark brown	15
15	Pronotum with two black spots on the disc. Dorsal part of left paramere strongly bent (Fig. 4 J–L)	*Gastroserica guangdongensis* Ahrens, 2000
–	Pronotum without spots on the disc. Dorsal part of left paramere slightly bent or straight	16
16	Frontoclypeal suture indistinctly impressed. Phallobase with a wider right paramere (Fig. 4 M–O)	*Gastroserica guizhouana* Ahrens, 2000
–	Frontoclypeal suture distinctly impressed. Phallobase with a more slender right paramere (Fig. 3 P–O)	*Gastroserica shaanxiana* Ahrens & Pacholátko, 2003
17	Even intervals of elytra black, but behind the middle, all the intervals black, with two brown spots at the apex of elytra. Interior apical angle of elytra with a strong seta (Fig. 1 J). Apex of right paramere sharp (Fig. 1 A–C)	*Gastroserica yunnanensis* sp. n.
–	Only the intervals next to the edge of elytra brown to black, without spots at the apex of elytra. Interior apical angle of elytra without strong setae. Apex of right paramere blunt (Fig. 4 G–I)	*Gastroserica bilyi* Ahrens, 2000
18	Left and right parameres fused ventrally (Fig. 2 M–O)	*Gastroserica marginalis* (Brenske, 1897)
–	Left and right parameres separated	19
19	Dorsal lobe of left paramere strongly curved ventrally. Apex of phallobasis with a short pointed apophysis at right side (Fig. 1 D–F)	*Gastroserica nigrofasciata* sp. n.
–	Dorsal lobe of left paramere short and straight, strongly curved ventrally. Apex of phallobasis without a short apophysis at right side (Fig. 3 D–F)	*Gastroserica yingi* Ahrens & Pacholátko, 2007

#### Gastroserica yunnanensis

Liu, Ahrens, Bai & Yang sp. n.

urn:lsid:zoobank.org:act:7385F187-5998-41D6-98BE-2331467C7D02

http://species-id.net/wiki/Gastroserica_yunnanensis

##### Type material.

 Holotype: 1♂”Caiyang River Nature Preserve, Pu’er, Yunnan, 28–29.8.2007, Shi Lei leg.” (LSSYU). Paratypes (1♂+1♀): 1♂”Caiyang River Nature Preserve, Pu’er, Yunnan, 28–29.8.2007, Shi Lei leg.”(LSSYU); 1♀”Caiyang River Nature Preserve, Yunnan, 28–29.8.2007, Li Jiahui leg.” (LSSYU).

##### Description.

**Figure 1.**
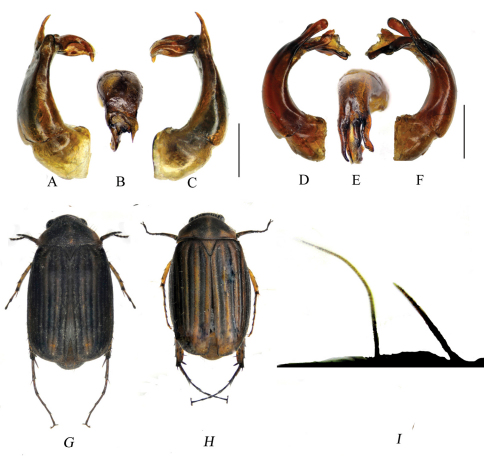
**A–C** *Gastroserica yunnanensis* sp. n. **D–F** *Gastroserica nigrofasciata* sp. n. **G** Habitus, *Gastroserica yunnanensis* sp. n. **H** Habitus, *Gastroserica nigrofasciata* sp. n. **I** Interior apical angle of elytra of *Gastroserica yingi*, with strong seta (the longer one). **A, D** aedeagus, left side lateral view **B, E** paramere, dorsal view **C,** **F** aedeagus, right side lateral view. Scale bar=1mm.

Length: 6.0–7.0 mm, length of elytra: 4.5–5 mm, width: 3.5–4.0 mm. Body oval,elytra brown, dorsal surface pale yellow to pale brown, densely covered with short, fine, adpressed setae and with moderately dense, long, erect setae interspersed, abdominal sternites dark brown to black. (Fig. 1 G)

**Figure 2.**
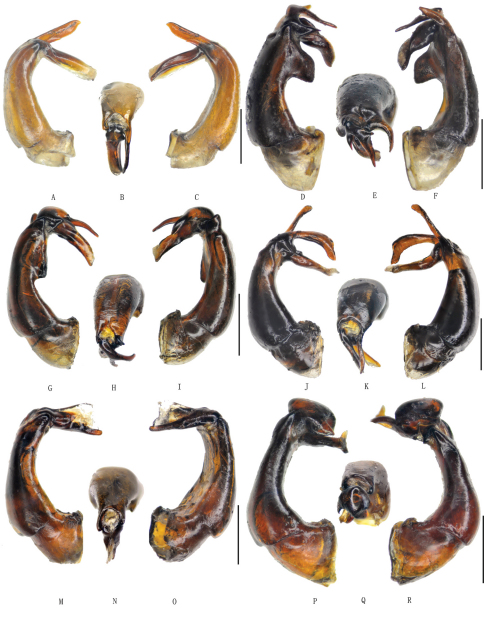
**A–C** *Gastroserica asulcata* Ahrens, 2000 **D–F** *Gastroserica hubeiana* Ahrens, 2000 **G–I** *Gastroserica sichuanana* Ahrens, 2000 **J–L** *Gastroserica kucerai* Ahrens & Pacholátko, 2003 **M–O** G. marginalis (Brenske, 1897) **P–R** *Gastroserica impressicollis* (Fairmaire, 1891). **A, D, G, J, M, P** aedeagus, left side lateral view **B, E, H, K, N, Q** paramere, dorsal view **C, F, I, L, O, R** – aedeagus, right side lateral view. Scale bar=1mm.

**Figure 3.**
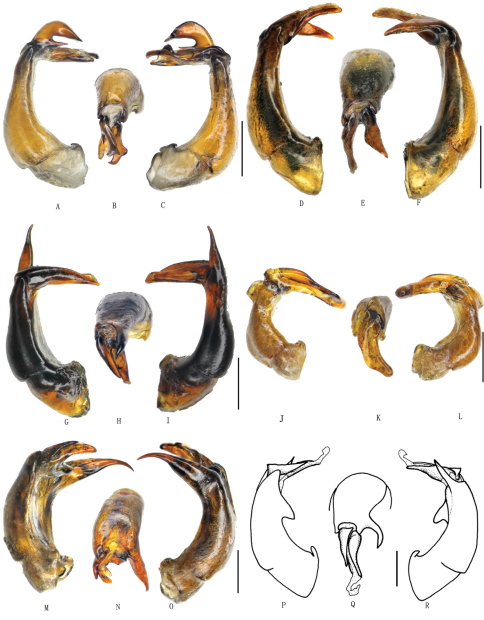
**A–C** *Gastroserica herzi* (Heyden, 1887) **D–F** *Gastroserica yingi* Ahrens & Pacholátko, 2007 **G–I** *Gastroserica sulcata* Brenske, 1897 **J–L** *Gastroserica haucki* Ahrens, 2000 **M–O** *Gastroserica fanjingensis* Ahrens, 2000 **P–R** *Gastroserica shaanxiana* Ahrens & Pacholátko, 2003. **A, D, G, J, M, P** aedeagus, left side lateral view **B, E, H, K, N, Q** paramere, dorsal view **C, F, I, L, O, R** aedeagus, right side lateral view. Scale bar=1mm. (**P–R** from Ahrens and Pacholátko 2003)

**Figure 4.**
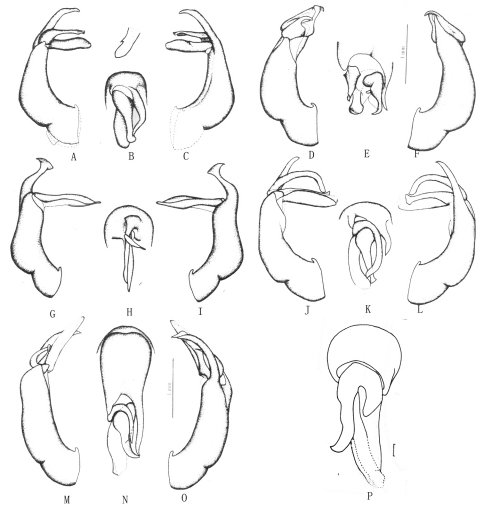
**A–C** *Gastroserica angustula* Brenske, 1897 **D–F** *Gastroserica nikodymi* Ahrens, 2000 **G–I** *Gastroserica bilyi* Ahrens, 2000 **J–L** *Gastroserica guangdongensis* Ahrens, 2000 **M–O** *Gastroserica guizhouana* Ahrens, 2000 **P** *Gastroserica bicolor* Nijima & Kinoshita, 1923. **A, D, G, J, M** aedeagus, left side lateral view **B, E, H, K, N, P** paramere, dorsal view **C, F, I, L, O** aedeagus, right side lateral view. Scale bar=1mm. (**A–O** from Ahrens 2000, and **P** from Nomura 1974)

Labroclypeus subrectangular and short, widest before apex, lateral margins straight, nearly parallel to each other and weakly convergent toward base, anterior angles broadly rounded, lateral border and ocular canthus produced into a distinct obtuse angle, anterior margin weakly reflexed, straight, surface weakly convex medially and moderately shiny, very coarsely and sparsely punctate, with several long, erect setae; frontoclypeal suture distinctly impressed and moderately curved, smooth area in front of eye slightly wider than long (1.5/1); ocular canthus moderately short and slender, finely and densely punctate. Frons completely black or only the part near the posterior portion brown, other parts of frons black, with coarse, dense punctures, with fine punctures irregularly interspersed, densely erectly setose. Eyes moderately large, ratio of diameter/ interocular width: 0.6. Antenna yellow, club yellow to brown, with ten antennomeres club with four antennomeres equal in length, the length of club a little longer than the remaining antennomeres combined. Mentum strongly elevated and flattened anteriorly.

Pronotum subrectangular, widest at anterior third, lateral margins strongly convergent anteriorly, before posterior angles weakly sinuate, anterior angles not produced and strongly rounded, almost obsolete, posterior angles moderately blunt and moderately produced outward, anterior margin almost straight, with a distinct and fine marginal line, basal margin moderately curved without marginal line, but two weak impressions at a quarter to the lateral margins; surface with moderately dense and fine punctures, with numerous minute setae, which are bentbackwards, with a longitudinal, straight, brown line in the middle and two black spots at sides of the disc; anterior and lateral borders setaceous; basal margin of hypomeron strongly produced ventrally, before base distinctly transversely sulcate. Scutellum nearly triangular, apex weakly rounded, with fine and dense punctures, medially smooth and weakly elevated, minute setae present in the punctures.

Elytra oblong, widest at middle, striae distinctly impressed and finely densely punctate, intervals weakly convex, with fine and sparse punctures that are almost concentrated along the striae, minutely setose in the punctures, odd intervals with single coarse punctures bearing each a strong erect seta, near the anterior margin, even intervals black, but behind the middle, all intervals black, with two brown spots at the apex of elytra; epipleural edge moderately strong, ending at the strongly convex external apical angle of elytra, epipleura densely setaceous, apical border chitinous, without short microtrichomes.

Ventral surface dull, with large and dense punctures, with dense, short setae, setae adpressed, metacoxa partly glabrous, laterally with fine adpressed setae, each abdominal sternite with an indistinct transversal row of coarse punctures bearing a short, strong seta between fine and dense punctation, all sternites with fine, short setae. Mesosternum between mesocoxae almost as wide as mesofemur, with numerous strong setae. Ratio of length of metepisternum/ metacoxa: 1/ 1.82. Pygidium long, apically produced and strongly convex, with fine and dense punctures bearing fine setae and a few robust punctures bearing robust setae, without smooth midline.

Legs pale yellow to brown, moderately slender and shiny; femora finely densely punctate and setose, with two longitudinal rows of setae; anterior edge of metafemur acute, lacking an adjacent serrated line, posterior margin weakly convex, with a few fine setae medially, ventrally weakly widened in apical half but not serrate, dorsally serrate, with short setae. Metatibia moderately broad, at middle convexly widened, ratio width/ length: 1/3.4, dorsally sharply carinate, with two groups of spines, the basal group at one third, apical one at two third of metatibia length, basally with a few single spines in punctures; lateral face longitudinally convex, with dense and moderately coarse punctures, some of them longitudinally impressed, ventral edge serrated, medial face not punctate and smooth, apex interiorly near tarsal articulation sharply truncate. Tarsomeres dorsally glabrous and finely punctate, ventrally with sparse, short setae, metatarsomeres dorsally with strong longitudinal impressions, ventrally with a strongly serrated ridge, laterally with a strong longitudinal carina, first metatarsomere as long as the following two tarsomeres combined and twice as long as the upper tibia spur. Protibia short, bidentate, protarsal claws symmetrical.

##### Aedeagus.

 Fig. 1 A–C.

##### Variation.

 Smooth area in front of eye wider than long (the rate from 1.5/1 to 1.7/1). Ratio of eye diameter / interocular width: (0.60–0.62). There are no brown spots at the end of elytra in some specimens, and after the middle, the odd intervals are still brown. Ratio of length of metepisternum/ metacoxa: (0.43~0.55).

##### Diagnosis.

 *Gastroserica yunnanensis* sp. n. is very similar to *Gastroserica bilyi* Ahrens, 2000, in shape of male genitalia and habitus. It maybe differentiated from *Gastroserica bilyi* by two brown spots at the end of elytra and sharp apex of right paramere.

##### Derivatio nominis.

 Named according to its provenience from Yunnan.

##### Distribution.

 Fig. 5.

**Figure 5.**
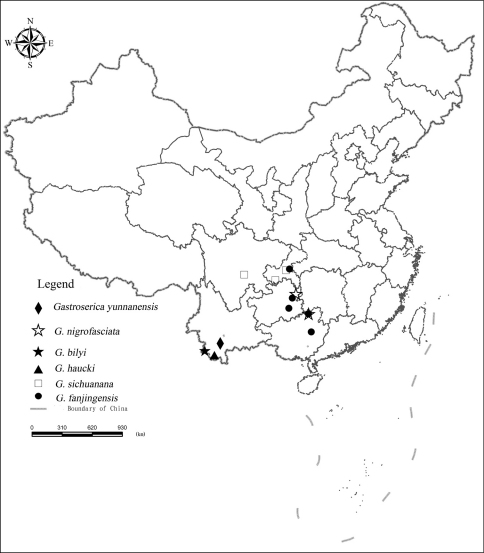
The distribution records of *Gastroserica yunnanensis*, *Gastroserica nigrofasciata*, *Gastroserica bilyi*, *Gastroserica haucki*, *Gastroserica sichuanana* and *Gastroserica fanjingensis* from China.

#### Gastroserica nigrofasciata

Liu, Ahrens, Bai & Yang sp. n.

urn:lsid:zoobank.org:act:2957A427-53AC-49A4-87E3-9FF333BF5D03

http://species-id.net/wiki/Gastroserica_nigrofasciata

##### Type material.

 Holotype: 1♂”Mt. Tianping Shan, Longsheng, Guangxi, 740 m, 9.6.1963, Wang Shuyong leg.” (IZAS). Paratypes (1♂+5♀♀): 1♂”Hongmaochong, Longsheng, Guangxi, 900 m, 10.6.1963, Shi Yongshan leg.” (ZFMK); 1♀” Hongmaochong, Longsheng, Guangxi, 900 m, 10.6.1963, Shi Yongshan leg.” (ZFMK); 1♀” Hongmaochong, Longsheng, Guangxi, 900 m, 10.6.1963, Wang Shuyong leg.” (IZAS); 1♀”Mt. Tianping Shan, Longsheng, Guangxi, 740 m, 4.6.1963, Wang Shuyong leg.” (IZAS); 1♀” Neicu River, Longsheng, Guangxi, 840 m, 7.6.1963, Wang Shuyong leg.” (IZAS); 1♀”Mt. Fanjing Shan, Jiangkou, Guizhou, 530 m, 12.7.1988, Wang Shuyong leg.” (IZAS).

##### Description.

Length: 5.6–8.0 mm, length of elytra: 4.1–5.1 mm, width: 3.2–4.1 mm. Body oval,elytra and dorsal surface both yellow, densely covered with short, fine adpressed setae and with moderately dense, long, erect setae interspersed (Fig. 1 H).

Labroclypeus subrectangular and short, widest at base, lateral margins straight and moderately divergent from anterior angles to base, anterior angles broadly rounded, lateral border and ocular canthus produced into a distinct obtuse angle, anterior margin weakly reflexed, straight, surface weakly convex medially and moderately shiny, very coarsely and sparsely punctate, with several long, erect setae; frontoclypeal suture distinctly impressed and strongly curved, smooth area in front of eye distinctly wider than long (1.8/1); ocular canthus moderately short and strong, finely and densely punctate. Frons with coarse, dense punctures, with fine punctures irregularly interspersed, densely erectly setose. Eyes moderately large, ratio of diameter/ interocular width: 0.63. Antenna brown, with ten antennomeres, club in male with four antennomeres, first joint of club slight shorter than the others, club slightly longer than the remaining antennomeres combined. Mentum elevated and flated anteriorly.

Pronotum rectangular, widest at half of length, lateral margins strongly convergent anteriorly, before posterior angles weakly sinuate, anterior angles not produced and strongly rounded, almost obsolete, posterior angles moderately blunt and weakly produced outward, anterior margin almost straight, with a distinct and fine marginal line, basal margin moderately curved without marginal line, and two weak impressions at quarter of width to the lateral margins; surface with moderately dense and fine punctures, with numerous minute setae, which are bentbackwards and two black spots at the middle, along the middle weakly medially impressed, with a weakly elevated transverse carina behind the middle; anterior and lateral borders setaceous; basal margin of hypomeron strongly produced ventrally, before base distinctly transversely sulcate. Scutellum nearly triangular, apex weakly rounded, with fine and dense punctures, medially smooth, minute setae present in the punctures.

Elytra oblong, widest at middle, striae distinctly impressed and finely densely punctate, intervals weakly convex, with fine and sparse punctures that are almost concentrated along the striae, minutely setose in the punctures, odd intervals with single coarse punctures bearing each a strong erect seta, even intervals brown to black; epipleural edge moderately strong, ending at the strongly convex external apical angle of elytra, epipleura densely setaceous, apical border chitinous, without short microtrichomes.

Ventral surface dull, with large and dense punctures and dense short setae, setae adpressed, metacoxa partly glabrous, laterally with fine adpressed setae, each abdominal sternite with indistinct transversal row of coarse punctures bearing each a short strong seta between fine and dense punctation, all sternites with fine, short setae. Mesosternum between mesocoxae almost as wide as mesofemur, with numerous strong setae. Ratio of length of metepisternum/ metacoxa: 1/ 2.0. Pygidium long, apically produced and strongly convex, with fine and dense punctures bearing fine setae and a few robust punctures bearing each a robust seta, without smooth midline.

Legs pale yellow to yellow brown, moderately slender and shiny, femora finely densely punctate and setose, with two longitudinal rows of setae; anterior edge of metafemur acute, lacking an adjacent serrated line, posterior margin weakly convex, with a few fine setae medially, ventrally weakly widened in apical half but not serrate, dorsally serrate, with short setae. Metatibia moderately broad, at middle convexly widened, ratio width/ length: 1/ 3.2, dorsally sharply carinate, with two groups of spines, the basal group at one third, apical one at two third of metatibial length, basally with a few single spines in punctures; lateral face longitudinally convex, with dense and moderately coarse punctures, some of them longitudinally impressed, ventral edge serrated; medial face not punctate and smooth, apex interiorly near tarsal articulation sharply truncate. Tarsomeres dorsally glabrous and finely punctate, ventrally with sparse, short setae, metatarsomeres dorsally with strong longitudinal impressions, ventrally with a strongly serrated ridge, laterally with a strong longitudinal carina, first metatarsomere as long as the following two tarsomeres combined and twice as long as the upper tibia spur. Protibia short, bidentate, protarsal claws symmetrical.

##### Aedeagus.

 Fig. 1 D–F.

##### Variation.

 Smooth area in front of eye wider than long (the rate from 1.8/1 to 2.0/1). Eyes weakly large, ratio of diameter/ interocular width: (0.60–0.67). Club a little shorter than the remaining antennomeres combined in female. Elytra from yellow to brown, with greenish metallic shine. Three intervals next to the lateral margins of elytra sometimes black. Ratio of length of metepisternum/ metacoxa: (1/ 1.82–2.).

##### Diagnosis.

 *Gastroserica nigrofasciata* sp. n. is in habitus very similar to *Gastroserica marginalis* (Brenske, 1897). It maybe differentiated from *Gastroserica marginalis* by the colour of elytra and dorsal surface, the presence of two long lateral apophyses at the apex of phallobasis, and the shape of parameres.

##### Derivatio nominis.

 From the Latin words “*nigro*-” and “*fasciata*” meaning black stripes.

##### Distribution.

 Fig. 5.

### New locality records

#### Gastroserica bilyi

Ahrens, 2000

http://species-id.net/wiki/Gastroserica_bilyi

Gastroserica bilyi  Ahrens, 2000: 113.

##### Material examined.

 1♂”Meng’a, Xishuangbanna, Yunnan, 1050 m, 20.5.1958, Pu Fuji leg.” (IZAS).

##### Notes.

This is a new record for China, the species was so far known only from Thailand and Laos.

##### Distribution.

 Fig. 5.

#### Gastroserica haucki

Ahrens, 2000

http://species-id.net/wiki/Gastroserica_haucki

Gastroserica haucki  Ahrens, 2000: 110.

##### Material examined.

 2♂♂”Menghun, Xishuangbanna, Yunnan, 1200–1400 m, 22.5.1958, Zhang Yiran leg.”(IZAS); 2♀♀” Menghun, Xishuangbanna, Yunnan, 1200–1400 m, 22.5.1958, Zhang Yiran leg.”(IZAS); 1♀”Menghun, Xishuangbanna, Yunnan, 1200–1400 m, 21.5.1958, Meng Xuwu leg.”(IZAS).

##### Notes.

This is a new record for China, the species was so far known only from Thailand and Laos.

##### Distribution.

 Fig. 5.

#### Gastroserica herzi

(Heyden, 1887)

http://species-id.net/wiki/Gastroserica_herzi

Serica herzi  Heyden, 1887: 264.
Microserica hertzi : Reitter 1896: 186.
Gastroserica herzi : Brenske 1897: 414; Ahrens 2000: 99.

##### Material examined.

 China: 3♂♂”Huangkeng’aotou, Jianyang, Fujian, 800–950m, 5.5.1960, Pu Fuji leg.” (IZAS); 1♂”San’gang, Chong’anxingcun, Fujian, 750m, 26.5.1960, Jiang Shengqiao leg.” (IZAS); 1♂, 1♀ ”Kuatun, Fukien China, 14.5.1946, leg. Tschung-Sen/ ex coll. V. Balthasar National Museum Prague, Czech Republic” (NMPC), 1♂, 1♀ ”Kuatun, Fukien China, 15.5.1946, leg. Tschung-Sen/ ex coll. V. Balthasar National Museum Prague, Czech Republic” (NMPC), 1♂ ”Kuatun, Fukien China, 16.5.1946, leg. Tschung-Sen/ ex coll. V. Balthasar National Museum Prague, Czech Republic” (NMPC), 2♂♂ “Kuatun (2300m) 27,40n. Br. 117,40o.L. J. Klapperich 8.5.1938, (Fukien)/ ex coll. V. Balthasar National Museum Prague, Czech Republic” (NMPC), 1♀ “Kuatun (2300m) 27,40n. Br. 117,40o.L. J. Klapperich 10.5.1938, (Fukien)/ ex coll. V. Balthasar National Museum Prague, Czech Republic” (NMPC), 1♀ “Kuatun (2300m) 27,40n. Br. 117,40o.L. J. Klapperich 12.5.1938, (Fukien)/ ex coll. V. Balthasar National Museum Prague, Czech Republic” (NMPC), 1♂ “Kuatun (2300m) 27,40n. Br. 117,40o.L. J. Klapperich 13.51938, (Fukien)/ ex coll. V. Balthasar National Museum Prague, Czech Republic” (NMPC), 1♂ “Kuatun (2300m) 27,40n. Br. 117,40o.L. J. Klapperich 25.5.1938, (Fukien)/ ex coll. V. Balthasar National Museum Prague, Czech Republic” (NMPC), 1♀ “Kuatun (2300m) 27,40n. Br. 117,40o.L. J. Klapperich 26.5.1938, (Fukien)/ ex coll. V. Balthasar National Museum Prague, Czech Republic” (NMPC), 1♂ “Kuatun (2300m) 27,40n. Br. 117,40o.L. J. Klapperich 1.6.1938, (Fukien)/ ex coll. V. Balthasar National Museum Prague, Czech Republic” (NMPC), 1♂ “Kuatun (2300m) 27,40n. Br. 117,40o.L. J. Klapperich 8.6.1938, (Fukien)/ ex coll. V. Balthasar National Museum Prague, Czech Republic” (NMPC), 1♂, 2♀♀ “Kuatun (2300m) 27,40n. Br. 117,40o.L. J. Klapperich 11.4.1938, (Fukien)/ ex coll. V. Balthasar National Museum Prague, Czech Republic” (NMPC), 2♂♂”Mt. Tianmu Shan, Zhejiang, 190m, 12.6.1936, O. Piel leg.” (IZAS); 2♂♂”E Mt. Tianmu Shan, Zhejiang, 190m, 12.6.1936, collector unknown.” (IZAS); 1♂”Lingtian, Lingchuan, Guangxi, 200m, 6.6.1984, Li Yuehua leg.” (IZAS); 1♂”Jinzhong Roud, Jinxiu, Guangxi, 1100m, 10.5.1999, Xiao Hui leg.” (IZAS); 1♂”Shiping, Fengdu, Sichuan, 610m, 3.6.1994, Yao Jian leg.” (IZAS); 1♂”Cangyuan, Yunnan, 990m, 16.5.1980, Shang Jinwen leg.” (IZAS); 1♂”Jianfengling, Hainan, 610m, 21.4.1983, Gu Maobin leg.” (IZAS); 1♂”Shiping, Fengdu, Sichuan, 610m, 2.6.1994, Li Wenzhu leg.” (IZAS); 1♀”Lingtian, Lingchuan, Guangxi, 200m, 6.6.1984, Wang Jizhen leg.” (IZAS); 1♀”Lingtian, Lingchuan, Guangxi, 200m, 6.6.1984, Luo Guifen? leg.” (IZAS); 2♀♀”Mt. Tianmu Shan, Zhejiang, 190m, 12.6.1936, O. Piel leg.” (IZAS); 2♀♀”Mt. Leigong Shan, Leishan, Guizhou, 1550m, 30.6.1988, Wang Shuyong leg.” (IZAS); 1♀”Mt. Tianping Shan, Longsheng, Guangxi, 740m, 4.6.1963, Wang Shuyong leg.” (IZAS); 1♀”Neicu Jiang River, Longsheng, Guangxi, 840m, 7.6.1963, Wang Shuyong leg.” (IZAS); 1♀”Mt. Fanjing Shan, Jiangkou, Guizhou, 530m, 12.7.1988, Wang Shuyong leg.” (IZAS). S. Korea: 1 ♂ “6.7.2010 Mudeungsan, Gwangju (Südkorea) leg. T. Kölkebeck” (ZFMK), 1 ♂ “8.7.2010 Mudeungsan, Gwangju (Südkorea) leg. T. Kölkebeck” (ZFMK), 3 ♂♂ “27.6.2010 Beomeosa, Busan (Südkorea) leg. T. Kölkebeck” (ZFMK), 2 ♂♂ “24.6.2010 Gwanggyosan, Suwon (Südkorea) leg. T. Kölkebeck” (ZFMK), 1 ♂ “23.6.2010 Suri-san, Gunpo-si, Geonggi-do (Südkorea) leg. T. Kölkebeck” (ZFMK), 1 ♀ “1.7.2010 Suri-san/ Ansan, Seoul (Südkorea) leg. T. Kölkebeck” (ZFMK), 1 ♂ “10.7.2010 Suri-san/ Ansan, Seoul (Südkorea) leg. T. Kölkebeck” (ZFMK).

##### Distribution.

 Fig. 7.

#### Gastroserica sichuana

Ahrens, 2000

http://species-id.net/wiki/Gastroserica_sichuana

Gastroserica sichuana Ahrens, 2000: 95.

##### Material examined.

 1♂”Baoguosi, Mt. Emei Shan, Sichuan, 550–750m, 2.5.1957, Wang Zongyuan leg.” (IZAS); 1♂”Shiping, Fengdu, Sichuan, 610m, 3.6.1994, Li Wenzhu leg.” (IZAS); 2♀♀”Shiping, Fengdu, Sichuan, 610m, 2–3.6.1994, Zhang Youwei leg.” (IZAS); 1♀”Nanmuyuan, Changshou, Sichuan, 450m, 9.6.1994, Zhang Youwei leg.” (IZAS); 3♀♀”Mt. Emei Shan, Sichuan, 800–1000m, 9–10.5.1957, Wang Zongyuan leg.” (IZAS); 1♀”Qiaoting, Wanxian, Sichuan, 1300m, 27.6.1974, Han Yinheng leg.” (IZAS).

##### Distribution.

 Fig. 5.

#### Gastroserica fanjingensis

Ahrens, 2000

http://species-id.net/wiki/Gastroserica_fanjingensis

Gastroserica fanjingensis Ahrens, 2000: 104.

##### Material examined.

 2♂♂”Huawangshanzhuang, Jinxiu, Guangxi, 600m, 20.5.1999, Li Wenzhu leg.” (IZAS); 1♂”Huawangshanzhuang, Jinxiu, Guangxi, 600m, 20.5.1999, Xiao Hui leg.” (IZAS); 1♂”Huawangshanzhuang, Jinxiu, Guangxi, 600m, 20.5.1999, Yang Xingke leg.” (IZAS); 2♂♂”Mt. TianpingShan, Longsheng, Guangxi, 740m, 4.6.1963, Wang Chunguang leg.” (IZAS); 1♂+1♀”Taiyuan, Pengshui, Sichuan, 800m, 12.7.1989, Yang Longlong leg.” (IZAS); 1♂”Mt. Fanjing Shan, Jiangkou, Guizhou, 530m, 12.7.1988, Wang Shuyong leg.” (IZAS); 2♀♀”Huawangshanzhuang, Jinxiu, Guangxi, 600m, 20.5.1999, Xiao Hui leg.” (IZAS); 1♀”Huawangshanzhuang, Jinxiu, Guangxi, 600m, 20.5.1999, Yang Xingke leg.” (IZAS).

##### Distribution.

 Fig. 5.

#### Gastroserica yingi

Ahrens, 2007

http://species-id.net/wiki/Gastroserica_yingi

Gastroserica yingi  Ahrens, 2007: 137.

##### Material examined.

 1♂”Mengzhe, Xishuangbanna, Yunnan, 1200m, 15.6.1958, Wang Shuyong leg.” (IZAS); 4♂♂”Mt. Tianping Shan, Longsheng, Guangxi, 740m, 4–5.6.1963, Wang Shuyong, Shi Yongshan, Wang Chunguang leg.” (IZAS); 1♀” Mengzhe, Xishuangbanna, Yunnan, 1200m, 15.6.1958, Wang Shuyong leg.” (IZAS).

##### Distribution.

 Fig. 6.

**Figure 6.**
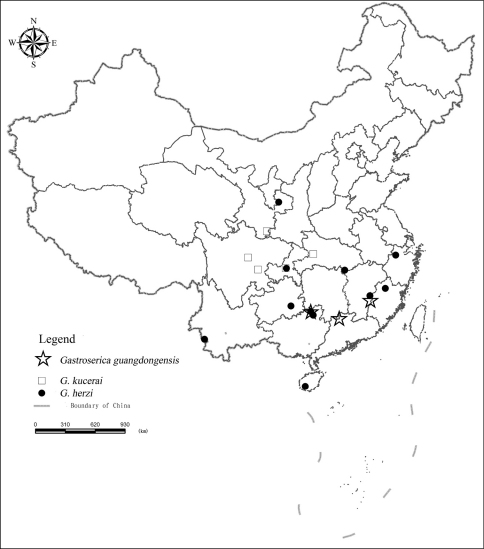
The distribution records of *Gastroserica guizhouana*, *Gastroserica nikodymi*, *Gastroserica bicolor*, *Gastroserica angustula*, *Gastroserica impressicollis* and *Gastroserica yingi* from China.

#### Gastroserica sulcata

Brenske, 1897

http://species-id.net/wiki/Gastroserica_sulcata

Gastroserica sulcata Brenske, 1897: 414.

##### Material examined.

 1♂”Mt. Tianping Shan, Longsheng, Guangxi, 740m, 4.6.1963, Wang Shuyong leg.” (IZAS); 1♂”Mt. Tianping Shan, Longsheng, Guangxi, 740m, 4.6.1963, Shi Yongshan leg.” (IZAS); 1♂”Shanmuhe Tree Farm, Yongshun, Hunan, 600–900m, 8.8.1988, Wang Shuyong leg.” (IZAS); 1♂”Mt. Fanjing Shan, Jiangkou, Guizhou, 530m, 12.8.1988, Wang Shuyong leg.” (IZAS); 1♂”Mt. Jiulian Shan, Jiangxi, 12.6.1975, Zhang Youwei leg.” (IZAS).

##### Distribution.

 Fig. 8.

#### Gastroserica kucerai

Ahrens & Pacholátko, 2003

http://species-id.net/wiki/Gastroserica_kucerai

Gastroserica kucerai Ahrens & Pacholátko, 2003: 2.

##### Material examined.

 3♂♂”Tianshidong, Mt. Qingcheng Shan, Sichuan, 1000m, 5.6.1979, Gao Ping leg.” (IZAS); 1♂”Qinghe Tree Farm, Kangxian, Gansu, 1400m, 8.7.1999, Yao Jian leg.” (IZAS); 3♀♀”Shiping, Fengdu, Sichuan, 610m, 3.6.1994, Yang Xingke leg.” (IZAS); 2♀♀”Shiping, Fengdu, Sichuan, 610m, 3.6.1994, Zhang Youwei leg.” (IZAS); 1♀”Longmenhe River, Xingshan, Hubei, 1350m, 14.7.1999, Chen Xiaolin leg.” (IZAS).

##### Distribution.

 Fig. 7.

**Figure 7.**
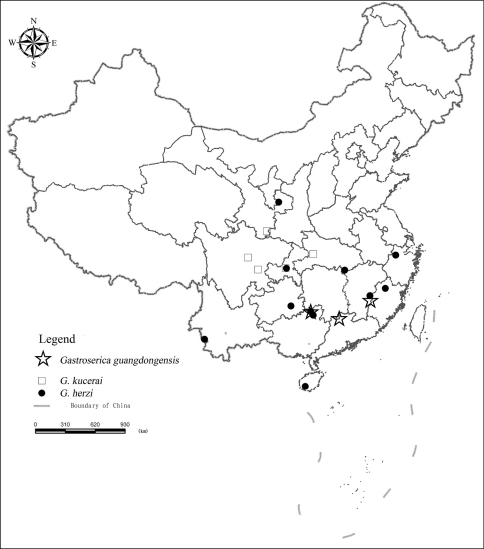
The distribution records of *Gastroserica guangdongensis*, *Gastroserica kucerai* and *Gastroserica herzi* from China.

#### Gastroserica impressicollis

(Fairmaire, 1891)

http://species-id.net/wiki/Gastroserica_impressicollis

Serica impressicollis  Fairmaire, 1891: 196.
Gastroserica impressicollis : Brenske 1897: 412, 416; Ahrens 2000: 97.

##### Material examined.

 8♂♂”Ku-ling, Jiangxi, 7–8.7.1935, O.Piel leg.” (IZAS); 2♂♂”Mt. Tianmu Shan, Zhejiang, 10–17.7.1935, O.Piel leg.” (IZAS); 1♂”Qingyin’ge, Mt. Emei Shan, Sichuan, 800–1000m, 11.6.1957, Lu Youcai leg.” (IZAS); 1♂”Qiaoting, Wanxian, Sichuan, 1300m, 27.6.1974, Han Yinheng leg.” (IZAS); 4♀♀” Baiyan, Longsheng, Guangxi, 1150m, 21.6.1963, Shi Yongshan leg.” (IZAS).

##### Distribution.

 Fig. 6.

#### Gastroserica hubeiana

Ahrens, 2000

http://species-id.net/wiki/Gastroserica_hubeiana

Gastroserica hubeiana Ahrens, 2000: 94.

##### Material examined.

 2♂♂”Longmenhe River, Xingshan, Hubei, 1300m, 10.5.1994, Zhang Youwei leg.” (IZAS); 1♂”Xinmaopeng, Mt. Tianmu Shan, Zhejiang, 1300m, 28.6.1957, collector unknown.” (IZAS); 1♂”Qingyin’ge, Mt. Emei Shan, Sichuan, 800–1000m, 28.5. 1957, Wang Zongyuan leg.” (IZAS).

##### Distribution.

 Fig. 8.

**Figure:**
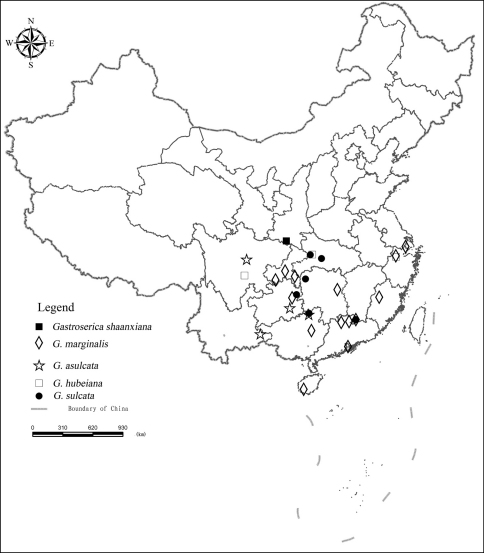
**Figure 8.** The distribution records of *Gastroserica shaanxiana*, *Gastroserica marginalis*, *Gastroserica asulcata*, *Gastroserica hubeiana* and *Gastroserica sulcata* from China.

#### Gastroserica guangdongensis

Ahrens, 2000

http://species-id.net/wiki/Gastroserica_guangdongensis

Gastroserica guangdongensis Ahrens, 2000: 90.

##### Material examined.

 2♂♂”Neicu Jiang River, Longsheng, Guangxi, 840m, 6.6.1963, Wang Shuyong leg.” (IZAS); 3♂♂”Mt. Tianping Shan, Longsheng, Guangxi, 740m, 4.6.1963, Shi Yongshan leg.” (IZAS); 1♂”Tongmu, Mt. Wuyi Shan, Fujian, 610m, 10.6.2001, Ge Siqin leg.” (IZAS).

##### Distribution.

 Fig. 7.

#### Gastroserica asulcata

Ahrens, 2000

http://species-id.net/wiki/Gastroserica_asulcata

Gastroserica asulcata  Ahrens, 2000: 79.

##### Material examined.

 2♂♂”Sanmen, Longsheng, Guangxi, 300m, 26.6.1963, Wang Chunguang leg.” (IZAS); 1♂”Taojiang River, Leishan, Guizhou, 1000m, 7.7.1988, Huang Fusheng leg.” (IZAS); 1♂”Taojiang River, Leishan, Guizhou, 1000m, 5.7.1988, Yang Longlong leg.” (IZAS); 1♂”Tianshidong, Mt. Qingcheng Shan, Sichuan, 1000m, 2.6.1979, Gao Ping leg.” (IZAS).

##### Distribution.

 Fig. 8.

#### Gastroserica marginalis

(Brenske, 1894)

http://species-id.net/wiki/Gastroserica_marginalis

Serica marginalis  Brenske, 1894 : 10.
Gastroserica marginalis  var. *puncticollis *Brenske, 1897: 413. Synonymized by Moser 1908: 331.
Gastroserica marginalis : Brenske 1897: 413; Ahrens 2000: 75.

##### Material examined.

 1♂”Shiping, Fengdu, Sichuan, 610m, 2.6.1994, Zhang Youwei leg.” (IZAS); 1♂”Nanmuyuan, Changshou, Sichuan, 450m, 9.6.1994, Li Wenzhu leg.” (IZAS); 1♂”Mt. Jiulian Shan, Jiangxi, 21.6.1975, Huang Fusheng leg.” (IZAS); 1♂”Mt. Jianfengling, Hainan, 8.4.1983, Gu Maobin leg.” (IZAS); 2♂♂”Xinmaopeng, Mt. Tianmu Shan, Zhejiang, 28.6.1957, Gu Maobin leg.” (IZAS); 1♂”Taoyuandong Nature Reserve, Yanling, Hunan, 631m, 5.7.2008, Yang Ganyan leg.” (IZAS); 1♂”Luoxiang, Jinxiu, Guangxi, 450m, 30.6.2000, Li Wenzhu leg.” (IZAS); 1♂”Mt. Tianjing Shan, Ruyuan, Guangdong, 18.6.1974, Chen Guanren leg. En-045835” (LSSYU); 1♂”Mt. Chebaling Shan, Shixing, Guangdong, 1000m, 26.1.1991, Wen Ruizhen leg. En-045872” (LSSYU); 2♀♀”Shiping, Fengdu, Sichuan, 610m, 2–3.6.1994, Zhang Youwei leg.” (IZAS); 1♀”Mt. Longxi Shan, Jiangle, Fujian, 26.6.1991, Yang Longlong leg.” (IZAS); 1♀”Wanfeng, Wulong, Hubei, 800m, 7.7.1989, Zhang Xiaochun leg.” (IZAS).

##### Distribution.

 Fig. 8.

## Supplementary Material

XML Treatment for Gastroserica yunnanensis

XML Treatment for Gastroserica nigrofasciata

XML Treatment for Gastroserica bilyi

XML Treatment for Gastroserica haucki

XML Treatment for Gastroserica herzi

XML Treatment for Gastroserica sichuana

XML Treatment for Gastroserica fanjingensis

XML Treatment for Gastroserica yingi

XML Treatment for Gastroserica sulcata

XML Treatment for Gastroserica kucerai

XML Treatment for Gastroserica impressicollis

XML Treatment for Gastroserica hubeiana

XML Treatment for Gastroserica guangdongensis

XML Treatment for Gastroserica asulcata

XML Treatment for Gastroserica marginalis
